# Opicapone in Parkinson’s disease: a real-world, multicenter, retrospective study to identify patient characteristics for sustained treatment benefit

**DOI:** 10.1007/s00702-025-03000-3

**Published:** 2025-08-31

**Authors:** Clelia Pellicano, Daniele Belvisi, Roberta Bovenzi, Matteo Costanzo, Maria I. De Bartolo, Francesca Sommaruga, Jessica Blandino, Nicola Modugno, Fabrizio Piras, Mariangela Pierantozzi, Alessandro Stefani, Giovanni Fabbrini, Tommaso Schirinzi

**Affiliations:** 1 Laboratory of Neuropsychiatry, Santa Lucia Foundation IRCCS, Rome, Italy; 2 Department of Human Neurosciences, Sapienza University of Rome, Rome, Italy; 3 Department of Neurology and Clinical Neurophysiology, IRCCS Neuromed, Pozzilli, Italy; 4 Unit of Neurology, Department of Systems Medicine, Tor Vergata University of Rome, Rome, Italy; 5 Department of Neuroscience, Istituto Superiore Di Sanità, Rome, Italy; 6 UOSD Parkinson, Tor Vergata University Hospital, Rome, Italy

**Keywords:** Catechol-*O-*methyltransferase inhibitor, Levodopa, Motor fluctuations, Opicapone, Parkinson’s disease, OFF time, End-of-dose wearing off

## Abstract

Fluctuations often complicate the course of Parkinson’s disease (PD). The third-generation catechol-*O*-methyltransferase inhibitor, opicapone, is safe and effective in patients with PD motor fluctuations, whether long-standing or earlier onset. Real-world data are needed to identify the most appropriate candidates for initiating and sustaining therapy with opicapone. To evaluate the long-term outcomes and identify predictors for safer and prolonged use of opicapone. This real-world, longitudinal, multicenter, retrospective study collected data on the demographics, medical history, motor subtype, motor severity, cognitive and psychiatric disorders at baseline when opicapone was introduced as add-on therapy and at the last follow-up. Discontinuation rates and main causes were recorded. Clinical features of patients who continued/discontinued were compared to identify predictors for drug discontinuation and define successful long-term outcomes. Of the 178 enrolled patients, 85% (35% of them female) continued opicapone for a mean of 30.2 ± 19.64 months. The remaining 15% (13.4% female) discontinued opicapone within 8.9 ± 11.30 months due to disabling dyskinesia, hypotension, gastrointestinal disorders, or psychiatric disturbances. Continuers were younger at baseline than those who discontinued, but clinical-demographic features did not predict continuing opicapone therapy. Continuers had significantly lower Hoehn and Yahr scale scores and fewer night-time OFF periods compared with discontinuers, whereas levodopa equivalent daily dose and burden of motor and neuropsychiatric complications were similar. Continued opicapone therapy was associated with less motor impairment and reduced night-time OFF. Safety and long-term tolerability were higher in younger patients with a tendency to milder motor severity.

## Introduction

Parkinson’s disease (PD) is a complex and heterogeneous neurodegenerative disorder responsible for a progressive and disabling syndrome, including motor and non-motor manifestations (Bloem et al. 2021). According to the Global Burden of Diseases (GBD) Collaborator Network, PD is the second most common neurodegenerative disease after Alzheimer’s disease, and rates of disability and death due to PD have been rapidly increasing (GBD Nervous System Disorders Collaborators 2024). It is estimated that approximately 11.8 million people globally were living with PD in 2021, an increase of 274% from 1990 (GBD Nervous System Disorders Collaborators 2024), reflecting an aging population with an increased life expectancy. Toxicological research has suggested that the rising rates of PD may be linked to increasing exposure to organochlorine pesticides and specific industrial solvents, notably trichloroethylene (Lock et al. 2013; Saeedi Saravi and Dehpour 2016).

In the early disease stages, dopaminergic drugs, including levodopa, provide optimal and stable motor control, accounting for a satisfactory recovery of motor signs (Bloem et al. 2021). Later in the disease course, patients often develop troublesome end-of-dose motor fluctuations and dyskinesias between periods of good motor response (“ON” periods) and periods of poor response or loss of response (“OFF” periods) due to fluctuations in levodopa plasma levels and the gradual loss of nigral dopaminergic neurons (LeWitt 2015; Olanow et al. 2020). End-of-dose motor fluctuations thus severely complicate the course of PD and impose a considerable social and economic burden (Rastgardani et al. 2020; Tanner 2020; Yang et al. 2020), accordingly, optimization of patient therapy is fundamental.

Opicapone (OPC, BIA 9-1067, Ongentys™, BIAL-Portela & Ca, S.A.) is a selective and reversible, peripherally-acting, third-generation catechol-*O*-methyltransferase (COMT) inhibitor designed as a once-daily oral add-on therapy to levodopa for the amelioration of motor fluctuations in patients with PD (Rocha et al. 2013). OPC has a high binding affinity for COMT and a slow disassociation rate for the OPC-enzyme molecular complex (Jenner et al. 2021; Jost 2022; Rocha et al. 2013). Previous studies have demonstrated the efficacy, safety, and tolerability of OPC in patients with PD, including those with long-standing and earlier-onset motor fluctuations (Antonini et al. 2023; Feldman and Margolesky 2023).

A post hoc analysis of the BIPARK-I and -II studies confirmed that, while OPC 50 mg was significantly more effective than placebo in reducing OFF time and increasing ON time across the whole trajectory of end-of-dose motor fluctuations, patients in the earlier stages of their PD course tended to experience enhanced benefit compared with those with later-stage disease (Rocha et al. 2021). Furthermore, the tolerability profile of OPC was even more favorable in patients at earlier versus later stages in their disease course (Rocha et al. 2022).

Although placebo-controlled clinical trials are the benchmark for assessing response to therapeutic intervention, real-world studies are essential to establish safety and effectiveness in the broader patient populations encountered in routine clinical practice. Furthermore, data from real-world studies are fundamental to identifying the most appropriate candidates for initiating and maintaining therapy with OPC. The currently available data from real-world settings support the findings from the pivotal clinical trials and show that patients with PD receiving OPC as an adjunct to levodopa-based therapy gain clinically meaningful improvements in motor fluctuations (Bacchin et al. 2024; Meira-Carvalho et al. 2021; Muñoz Ruiz et al. 2020; Reichmann et al. 2020). Treatment-emergent adverse events reported in published real-world studies align with those observed in clinical trials and are primarily of mild-to-moderate severity.

However, real-world data on OPC remain limited. Therefore, the objective of this study was to evaluate the long-term outcomes of OPC therapy and identify predictors for safe and prolonged use of the drug.

## Methods

We performed a real-world, longitudinal, multicenter, retrospective study of patients with PD treated at the Movement Disorders Outpatient Clinic of the Sapienza University of Rome, Tor Vergata University Hospital, and Santa Lucia Foundation IRCCS, Italy. Patients were eligible for inclusion if they were ≥ 18 years old, had been diagnosed with PD according to international criteria (Postuma et al. 2015), and were receiving OPC as an add-on to levodopa therapy. Exclusion criteria were a diagnosis or suspicion of atypical/secondary parkinsonism or overt dementia (Mini Mental State Examination score of < 10).

Data on the patients’ demographics, medical history, motor subtype (tremor predominant, rigid-akinetic, mixed) according to Stebbins et al. 2013 (Stebbins et al. 2013), motor symptom severity, cognitive and/or psychotic symptoms (such as delusions and hallucinations), as well as anxiety disorders, and therapy were collected from their clinical charts at baseline (T0), when OPC was introduced as add-on therapy, and at the last follow-up visit (T1).

The patient cohort was stratified into those who continued OPC (PDcont) and those who discontinued OPC between T0 and T1 (PDdisc), and the discontinuation rate and reasons for discontinuation recorded. Clinical characteristics of patients in the PDcont and PDdisc groups were compared to identify predictors of OPC discontinuation and to define outcomes for those who stayed on OPC long term.

The study adhered to the principles of the Declaration of Helsinki and subsequent amendments. The relevant ethical committees approved the design of the study. As the patients received treatment as part of routine clinical practice, there was no specific requirement for informed consent to participate in the study, and all patient data were anonymized.

### Statistical analysis

The analysis included data collected at T0 and T1. Data from these time points were summarized using descriptive statistics. Depending on the normality of the distribution, continuous variables are summarized as means (± standard deviation) or medians and compared by the independent-sample t-test or Wilcoxon rank-sum test. Categorical variables are presented as numbers and percentages and were compared using the Chi-square test or Fisher’s exact test. A logistic regression analysis was utilized to screen the possible risk factors related to discontinuation of OPC. A two-tailed *p* < 0.05 was considered statistically significant.

The analyses were conducted using SPSS software version 29.0 (IBM, Armonk, New York, USA).

## Results

The observation period covered by the clinical charts was from 2018 to 2024. The demographic, clinical, and therapeutic data of patients are reported in Table 1. One hundred and seventy-eight patients (34.8% female), with a mean age of 64.1 ± 9.2 years, were enrolled in the study (Table 1). At baseline, male patients were younger (*p* = 0.03), had milder motor severity (*p* = 0.019), as expressed by the mean Hoehn and Yahr (H&Y) stage, and were less likely to have levodopa-induced dyskinesia (*p* = 0.009) but had a higher rate of psychiatric disorders than female patients (*p* = 0.002; see Online Resource 1).

**Table 1:** Demographic and clinical characteristics in the full analysis set (*N* = 178)

Characteristic	Value
*Sex, n (%)*	
Male	116 (65.2)
Female	62 (34.8)
*Age, years, mean* ± *SD (range)*	
At baseline (T0)^a^	64.1 ± 9.2 (37–85)
Males	63.1 ± 9.2 (37–83)
Females^b^	66.0 ± 8.8 (46–85)
At disease onset	56.7 ± 10.01 (31–84)
*Motor phenotype, n (%)*	
Tremor dominant	63 (35.4)
Rigid-akinetic	93 (52.2)
Mixed	22 (12.4)
*Other anti-Parkinsonian drugs at baseline, n (%)*	
MAO-i	136 (76.4)
Dopamine agonist	92 (51.7)
Other COMT-i	15 (8.4)

*COMT*-*i* catechol-*O*-methyltransferase inhibitor, *MAO*-*i* monoamine oxidase inhibitor, *SD* standard deviation

^a^Data available for 167 patients (109 male and 58 female). ^b^Independent samples *t*-test, *p* = 0.051 vs males

**Fig. 1:**
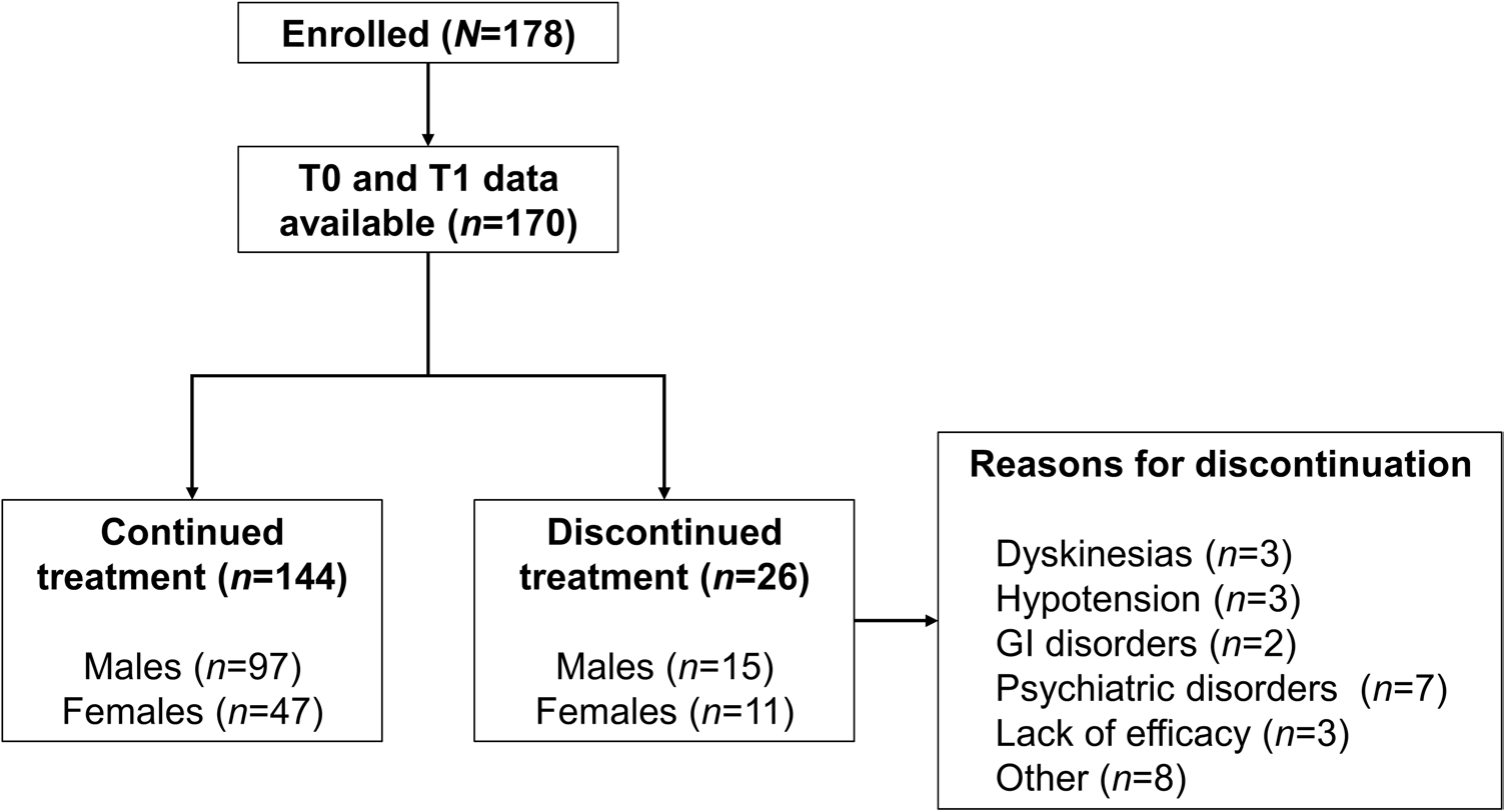
Patient flow through the study. *GI* gastrointestinal, *T0* baseline, *T1* follow-up

Figure 1 shows the flow of patients throughout the study. The mean duration of OPC therapy was 26.9 ± 20.11 (median 24.0, 0–108) months based on the available data (*n* = 170). In the cohort of 170 patients for whom data relating to the continuation/discontinuation of OPC were available, 144 (84.7%) continued OPC up to T1 for a total of 30.2 ± 19.64 months, whereas 26 (15.3%) discontinued OPC within 8.9 ± 11.30 months of treatment introduction (*p* < 0.001; Table 2). Eight patients were lost to follow-up, but they were included in the total number of patients enrolled at baseline, to better define the profile of patients receiving OPC as add-on treatment in this specific social and geographic context. There was a significant difference in the duration of OPC therapy between males (mean 29.2 ± 20.26; median 24.0, 0–108 months) and females (mean 22.6 ± 9.26; median 18.0, 0.5–72 months, *p* = 0.042).

**Table 2:** Time-point analysis of patients who continued or discontinued opicapone during 3 years of follow-up (*N* = 170)

	Baseline (T0)			Follow-up (T1)		
	Continued (*n* = 144)	Discontinued (*n* = 26)	*p* value	Continued (*n* = 144)	Discontinued (*n* = 26)	*p* value
*Sex, n (%)*						
Male	97 (67.4)	15 (57.7)	0.339^a^	97 (67.4)	15 (57.7)	0.339^a^
Female	47 (32.6)	11 (42.3)	–	47 (32.6)	11 (42.3)	–
Age, years, mean ± SD	63.3 ± 9.00	67.6 ± 9.21	**0.032**^**b**^	66.0 ± 9.12	70.5 ± 9.37	**0.021**^**b**^
Age at onset, years, mean ± SD	55.7 ± 9.67	60.9 ± 10.11	**0.015**^**b**^	–	–	–
Disease duration, years, mean ± SD	7.3 (4.35)	7.3 (4.23)	0.957^b^	10.2 ± 4.47	9.8 ± 4.03	0.689^b^
Treatment duration, months, mean ± SD	–	–	–	30.2 ± 19.64	8.9 ± 11.30	** < 0.001**^**b**^
*Duration of follow-up, mean* ± *SD*						
Male	–	–	–	–	29.2 ± 20.26	0.042
Female	–	–		–	22.6 ± 9.26	
*Phenotype, n (%)*						
Tremor dominant	54 (85.7)	9 (14.3)	0.939^a^	54 (85.7)	9 (14.3)	0.939^a^
Rigid-akinetic	76 (84.4)	14 (15.6)		76 (84.4)	14 (15.6)	
Mixed	14 (82.4)	3 (17.6)		14 (82.4)	3 (17.6)	
H&Y stage, mean ± SD	2.4 ± 0.42	2.6 ± 0.34	0.056^c^	2.5 ± 0.48	2.9 ± 0.79	**0.002**^**c**^
Duration from MF diagnosis to OPC initiation, years, mean ± SD	–	–	–	3.0 ± 3.28	2.3 ± 2.51	0.342^b^
*Complications, n (%)*						
Night-time OFF	86 (64.2)	12 (50.0)	0.187^a^	58 (40.3)	17 (65.4)	**0.018**^**a**^
Dyskinesia (LID)	67 (49.6)	13 (54.2)	0.682^a^	97 (67.4)	15 (61.5)	0.563^a^
Cognitive disorders	54 (40.0)	10 (41.7)	0.878^a^	65 (45.1)	9 (34.6)	0.305^a^
Psychiatric disorders^d^	24 (17.8)	6 (25.0)	0.405^a^	28 (19.4)	8 (30.8)	0.193^a^
ICD	12 (8.9)	2 (8.3)	0.929^a^	9 (6.2)	3 (11.5)	0.333^a^
LEDD, mg/day, mean ± SD	669 ± 225.3	658 ± 188.5	0.835^b^	768 ± 314.3	758 ± 262.1	0.880^2^
Concomitant use of MAO, *n* (%)	98 (72.6)	17 (70.8)	0.859^a^	86 (59.7)	14 (50.0)	0.355^a^
Concomitant use of DA, *n* (%)	80 (59.3)	9 (37.5)	**0.048**^**a**^	70 (48.6)	8 (30.8)	0.093^a^

Bold refers to significant p values

The percentages were calculated using the total number of subjects with available data as the denominator

*DA* dopamine agonist, *F* female, *H&Y* Hoehn and Yahr, *ICD* impulse control disorder, *LEDD* levodopa-equivalent daily dose, *LID* levodopa-induced dyskinesia, *M* male, *MAO* monoamine oxidase inhibitor, *MF* end-of-dose motor fluctuations, *OPC* opicapone, *SD*standard deviation

^a^Chi-squared test

^b^Independent samples *t*-test

^c^Wilcoxon test

^d^Psychotic symptoms (such as delusions or hallucinations) and anxiety disorders

Discontinuation was reported in 20% of females (11/54) and 14% of males (15/105) (*p* > 0.05). There were sex-specific differences in the causes of discontinuation: hypotension and psychiatric disorders were numerically more prevalent in males while disabling dyskinesia and lack of efficacy were reported more frequently in females (Table 3). Gastrointestinal disorders led to OPC discontinuation in one male and one female. Eight patients (6 males and 2 females) discontinued for other reasons, including drowsiness, malaise, and postural instability (Table 3).

**Table 3:** Reasons for discontinuation in patients who discontinued opicapone (*N* = 26)

Cause for discontinuation	Patients (%)	Time from T0 to discontinuation, months, mean ± SD
*Any*		8.9 ± 11.30
Males	15/105 (57.7)	
Females	11/54 (42.3)	
*Dyskinesia*		13.0 ± 9.64
Males	0 (0.0)	
Females	3 (100.0)	
*Hypotension*		4.8 ± 6.33
Males	2 (66.7)	
Females	1 (33.3)	
*Gastrointestinal disorders*^*a*^		2.3 ± 0.35
Males	1 (50.0)	
Females	1 (50.0)	
*Psychiatric disorders*^*b*^		11.2 ± 16.96
Males	6 (85.7)	
Females	1 (14.3)	
*Lack of efficacy*		6.7 ± 0.58
Males	0 (0.0)	
Females	3 (100.0)	
*Other*^*c*^		9.3 ± 11.70
Males	6 (75.0)	
Females	2 (25.0)	

*SD* standard deviation, *T0* baseline

^a^Diarrhea or nausea

^b^Psychotic symptoms (such as delusions or hallucinations) and anxiety disorders

^c^Drowsiness, malaise, or postural instability

At baseline (T0), PDcont patients were younger than PDdisc patients (*p* = 0.032), and continuing OPC was also associated with lower age at disease onset (*p* = 0.015; Table 2).

Milder motor severity at baseline and concomitant use of a dopamine agonist were approaching or marginally significantly associated with the continuation of OPC (*p* = 0.056 and *p* = 0.048, respectively). However, other clinical and demographic features, including sex, motor severity, neuropsychiatric comorbidity, and concomitant monoamine oxidase inhibitor use, did not differ significantly between the two groups (Table 2).

At follow-up (T1), H&Y stage and the prevalence of night-time OFF periods were significantly lower in PDcont patients (*p* = 0.002 and *p* = 0.018, respectively; Table 2). However, the levodopa equivalent daily dose (LEDD) and the burden of motor and neuropsychiatric complications were similar.

A logistic regression analysis using age, sex, clinical site, age at onset, disease duration, H&Y stage, night-time OFF, dyskinesia, cognitive disorders, and psychiatric disorders as variables found that no variables were predictive of discontinuation of OPC (data not shown).

## Discussion

This real-world, multicenter, retrospective study covering most of the period that OPC has been available for PD in Italy showed that the drug is substantially well tolerated, with a low rate of adverse event-related discontinuations. A younger age and possibly milder motor severity among patients receiving OPC add-on therapy were associated with greater safety and long-term tolerability. Sex did not affect the outcome, with the discontinuation rate similar in females and males (20% vs 14%), although the data suggested that reasons for discontinuation may differ between females and males. After the same follow-up time, lasting about three years, patients continuing OPC treatment had a lesser degree of motor impairment and reduced night-time OFF than those who discontinued earlier, although with a similar LEDD.

The management of patients with levodopa-associated complications is challenging. There is broad agreement about the clinical utility of OPC as an effective adjunctive option in treating end-of-dose motor fluctuations in PD, to reduce early morning and night-time akinesia, reduce OFF time, and increase ON time, while improving patient quality of life, regardless of the presence of dyskinesia (Antonini et al. 2023; Hauser et al. 2024). A recent multicenter study of treatment patterns in PD found that OPC was the COMT inhibitor most frequently prescribed as oral adjunctive therapy in patients with advanced PD in Italy (Stocchi et al. 2024). A post hoc analysis of clinical trials showed that OPC reduced OFF time before, during, and after night-time sleep periods, particularly for patients who were awakening in an OFF state during the night (Hauser et al. 2024). Moreover, OPC was more effective than increasing the levodopa dose for reducing OFF time in patients with early signs of wearing off (Ferreira et al. 2024). This supports a strategy of starting OPC treatment in the early stages of motor fluctuations, when the patient’s endogenous buffering capacity remains relatively intact, and they can gain the benefit of increased levodopa plasma levels associated with continuing and effective stimulation (Antonini et al. 2023; Bacchin et al. 2024; Olanow et al. 2020). Notably, a large real-world study showed that adding OPC as an adjunct to levodopa in routine clinical practice significantly improved the perceptions of patients and clinicians about the patients’ global PD condition compared with perceptions in the pre-OPC baseline period (Reichmann et al. 2020).

Pooled analyses of clinical trials and real-world experience demonstrate that OPC is well tolerated, with generally similar tolerability profiles regardless of age (Scott 2021). Introducing adjunctive OPC to treat end-of-dose fluctuations has also been shown to have a more favorable tolerability profile when used earlier rather than later in the disease course (Rocha et al. 2022). The most common treatment-related adverse event reported in clinical trials of OPC versus placebo has been dopaminergic dyskinesia (17.7% vs. 6.2%) (Ferreira et al. 2019). Interestingly, the post hoc analysis of clinical trials showed that dyskinesia, and other dopaminergic-related adverse events (nausea, hallucination, orthostatic hypotension, and vomiting), were lower for patients in earlier versus later stages of their disease course (Rocha et al. 2022).

Our data suggest that sex-specific differences may be associated with the causes of OPC discontinuation, although these differences did not affect the clinical outcome. Although, in our real-world study, there was an increase in dyskinesia between baseline and follow-up, troublesome dyskinesia leading to discontinuation occurred in only three patients (all female), whereas males discontinued for other reasons. These data are probably explained by the greater sensitivity of female patients to dopaminergic stimulation and the contribution of sex hormones to the motor response of patients (Bovenzi et al. 2025, 2023b; Conti et al. 2022). Other reasons include the different sex-related profiles of non-motor symptoms and non-motor fluctuations that may influence side effects (Bovenzi et al. 2024; Donzuso et al. 2023).

Patients in our study were also significantly more likely to discontinue OPC therapy if they were older at baseline, had an older age at disease onset, and were not taking concomitant dopamine agonists. These results probably derive from the different pathology of motor circuits characterizing patients with earlier age at onset, while the data on dopamine agonists may follow the practice of avoiding that class of drugs in older patients (Bovenzi et al. 2023a). There was also a trend towards a higher rate of discontinuation among patients with worse motor severity at baseline, but motor severity did not reach statistical significance as a predictor of prolonged use of OPC.

The present findings are in line with data from a recent real-world Italian investigation that found OPC to have good tolerability and a favorable safety profile, with disease stage and duration of motor fluctuations predictive of drug tolerability (Bacchin et al. 2024). In that study, an H&Y score ≥ 2.5 and motor fluctuations for ≥ 12 months were the leading factors associated with OPC therapy discontinuation caused by dopamine-related adverse events. Other researchers have identified age at PD onset, disease duration, motor symptom severity, and use of levodopa with concomitant dopamine agonists as factors predictive of the safety and tolerability of OPC (Muñoz Ruiz et al. 2020; Rocha et al. 2022).

The overall discontinuation rate in our study was just 15.3%; of the 26 patients who discontinued OPC, 15 were male (57.7%) and 11 were female (42.3%). By comparison, the OPC discontinuation rate in the recent real-world Italian study described above was 30% overall and 27% due to adverse events during 2 years of follow-up (Bacchin et al. 2024). This is higher than the 8.4% rate of discontinuation related to treatment-emergent adverse events reported in a meta-analysis of long-term (≥ 6 months) extension and open-label regulatory studies (Xie et al. 2022). However, it is closer to the OPC discontinuation rate reported in other real-world studies in Spain (López-Ariztegui et al. 2021) and Portugal (Meira-Carvalho et al. 2021), which were 23% and 20.8%, respectively. The differences in the discontinuation rate among studies can be explained by differences in disease stage among study populations; in fact the above-mentioned studies suggested that the drug discontinuation rate due to dopaminergic-related adverse events was lower in patients with a less advanced disease state and mild motor fluctuations (Bacchin et al. 2024; López-Ariztegui et al. 2021).

Finally, we observed that at the end of the follow-up period, lasting about three years, patients who continued OPC had milder motor severity, measured as H&Y stage, and lower prevalence of night-time OFF than those who discontinued earlier, although with a similar LEDD, suggesting that OPC may optimize and enhance the response to dopaminergic treatment over time (Bacchin et al. 2024; Muñoz Ruiz et al. 2020; Rocha et al. 2022).

Younger age at disease onset, female sex, disease duration, severity of PD at levodopa initiation, higher levodopa dose, and treatment duration are risk factors for the development of end-of-dose motor fluctuations (Kostic et al. 1991; Kostíc et al. 2002; Patel and Kompoliti 2023; Schrag et al. 1998). The design of our study took these factors into consideration, thus allowing us to account for several potential confounders that could have influenced the results. In addition, the study followed patients over an extended period and involved a substantial number of patients.

We acknowledge that our study has some limitations, including those inherently related to the retrospective design. Moreover, the small sample size limits generalizability, particularly with regard to the effect of sex on the discontinuation of OPC. Although the data were collected prospectively, some data were lacking, including those related to comorbidities, and weight-adjusted doses of levodopa after the introduction of OPC. Identifying predictive factors is complicated by the interdependence of clinical variables such as H&Y scale score, disease duration, and duration of motor fluctuations, and only a limited number of factors with possible predictive value were identified in our study. As was to be expected, there was a higher prevalence of male patients in the study, who were, on average, younger than females. Nevertheless, the results show that younger patients with milder motor severity are more likely to continue and benefit from treatment with OPC.

## Conclusions

OPC is a valuable adjunctive therapy to levodopa for improving motor control in patients with PD, with proven efficacy and a favorable tolerability profile. Continuation of OPC treatment is associated with a lesser degree of motor impairment and reduced prevalence of night-time OFF. Our findings support the data that OPC is especially useful in patients with a less advanced disease stage, and the safety and long-term tolerability of OPC are higher in younger patients with milder motor severity. The earlier OPC is introduced into the therapeutic strategy for managing motor fluctuations, the better the benefit-risk ratio for the drug.

## Data Availability

The datasets used and/or analyzed during the current study are available from the corresponding author upon reasonable request.
